# Efficacy and safety of Xiaochaihu decoction for subacute thyroiditis: A protocol of systematic review and meta-analysis

**DOI:** 10.1097/MD.0000000000023011

**Published:** 2020-11-13

**Authors:** Linzhi Li, Rensong Yue, Lihong Zeng, Shengnan Wang, Wuhui Zhuo, Yingying Sun

**Affiliations:** a Hospital of Chengdu University of Traditional Chinese Medicine; b Chengdu University of Traditional Chinese Medicine, Chengdu, China.

**Keywords:** meta-analysis, protocol, subacute thyroiditis, systematic review, Xiaochaihu decoction

## Abstract

**Background:**

Subacute thyroiditis (SAT) is a transient and self-limiting inflammatory thyroid disease. There is no clear evidence for specific etiology, but it is generally thought to occur after viral infection. Characteristics of SAT include severe pain of the anterior neck, enlarged firm thyroid, disordered thyroid function, elevated erythrocyte sedimentation rate (ESR) and C-reactive protein (CRP), typical ultrasound findings (hypoechoic areas) and low thyroid uptake of radioactive iodine or technetium-99 m because of the destructive etiology of the hyperthyroidism. Evidences showed Xiaochaihu decoction (XCHD) has a significant effect on improving the symptoms of SAT patients. The purpose of this study is to assess the safety and effectiveness of XCHD for patients with SAT.

**Methods and analysis:**

The literature that has been identified via searching 6 Chinese electronic databases and eight English electronic databases from inception to September 21, 2020 will be included in the study. Research selection, data extraction as well as research quality assessment will be completed by 2 experienced researchers independently. The primary outcome is remission rate. Data analysis will be conducted by the RevMan 5 software, and GRADE will help to assess the level of evidence. The heterogeneity of data will be investigated by a heterogeneity *x*^*2*^ test, as well as the Higgins *I*^2^ test. A subgroup analyses and sensitivity analysis will be conducted to explore the sources of heterogeneity.

**Results:**

The results of this study will be published in a peer-reviewed journal.

**Conclusion:**

This study will draw a conclusion about whether XCHD is safe and effective in treating SAT on the basis of evidence-based medicine. This conclusion will provide areliable scientific evidence for the alternative treatment for the management of SAT.

**OSF registration number:**

https://osf.io/8hbue.

## 1 Introduction

Subacute thyroiditis (SAT), also named subacute granulomatous or subacute painful thyroiditis, is a transient and self-limiting inflammatory thyroid disease.^[1]^ With the increase of work pressure and the acceleration of the pace of life in modern society, the incidence of SAT is on the rise every year.^[2]^ A research shows the prevalence of SAT is nearly 5% in the population with abnormal thyroid function.^[3]^ SAT often occurs in women aged 20 to 60 and the incidence is 5 times higher in women than in men.^[4,5]^ There is no explicit evidence for specific etiology, but it is generally believed that it occurred after viral infection.^[6,7]^ Characteristics of SAT include the following: severe pain of the anterior neck, enlarged firm thyroid, disordered thyroid function, elevated C-reactive protein (CRP) and erythrocyte sedimentation rate (ESR), typical ultrasound findings (hypoechoic areas) and low thyroid uptake of radioactive iodine or technetium-99 m caused by the destructive etiology of the hyperthyroidism.^[8–11]^ Other symptoms, such as fever, malaise, fatigue, myalgia, and radiating pain to the pharynx and jaw, are also observed.^[9,12,13]^

The initial treatment aims to reduce the inflammation and relieve the clinical symptoms. However, no etiological treatment has been established yet.^[4]^ Symptomatic treatment, including nonsteroidal anti-inflammatory drugs (NSAIDs) and glucocorticoids based on the severity of symptoms, is recommended by the American Thyroid Association (ATA) and the Chinese Endocrinology Association.^[14,15]^ Although this therapy can alleviate the clinical symptoms of the SAT patients, the side effects associated with the glucocorticoids may occur. Long-term glucocorticoid therapy can increase blood glucose, blood pressure, blood lipid levels, body weight and cause disorders of bone metabolism, Cushingoid features and abdominal discomfort.^[16,17]^ Those adverse events lead to high treatment discontinuation rate, unsatisfying curative efficacy, and a high recurrence rate.^[18]^ Although patients receive comprehensive treatment in time, unfortunately, some patients will ultimately develop permanent hypothyroidism and require long-term thyroid hormone-replacement treatment.^[9]^

Chinese herbal medicine is one of the most important parts of Traditional Chinese Medicine (TCM), which has been used for thousands of years. Accumulated evidences proved that Chinese herbal medicine can properly alleviate the clinical symptoms of the SAT patients.^[19–21]^ Xiaochaihu decoction (XCHD), a prescription with thousands of years of clinical experience in China, is still widely in use today. Xiaochaihu decoction, composed of Chaihu (Radix Bupleuri), Banxia (Pinellia ternata), rensheng (Radix Ginseng), huangqin (Radix Scutellariae), shengjiang (fresh ginger), gancao (Radix Glycyrrhizae), and dazao (Fructus Jujubae), has proven to be effective in treating subacute thyroiditis, yet its real efficacy and safety are not well documented.^[22–29]^ In order to certify this point, a protocol for a systematic review and meta-analysis of XCHD for subacute thyroiditis will be performed.

## 2 Methods

### 2.1 Study registration

The protocol has been registered on OSF (Open Science Framework) platform and the registration number is https://osf.io/8hbue. This protocol was written in accordance with the statement guidelines of Preferred Reporting Items for Systematic Reviews and Meta-Analyses Protocols (PRISMAP) checklist.^[30]^

### 2.2 Inclusion and exclusion criteria for study selection

All randomized controlled trials (RCTs) about XCHD (including Chinese patent medicine for XCHD) will be included. The studies of non-RCTs, quasi-RCTs, animal experiments, case series and reviews will be excluded. The language is limited to Chinese and English.

### 2.3 Participants

Patients clinically diagnosed with SAT. No age, region, gender, ethnicity, or education restriction.

### 2.4 Interventions

#### 2.4.1 Experimental interventions

Interventions in the experimental group is CXHD including decoction, patent medicine and granules.

#### 2.4.2 Control interventions

Interventions in the control group include placebos or drugs that have been proven effective in the treatment of SAT.

### 2.5 Types of outcome measures

#### 2.5.1 Primary outcomes

The primary outcome is symptom remission rate. All the uncomfortable symptoms will be evaluated, such as thyroid pain, tenderness, thyroid enlargement, fever and other clinical symptoms.

#### 2.5.2 Secondary outcomes

Secondary outcomes include: Changes of serum antibodies: thyroid microsomal antibody and anti-thyroglobulin antibodies; time period of returning normal ESR and CRP; the recurrence rate after treatment (1 month,3 months, 6 months,1 year); evaluation of adverse events.

## 3 Study search

Electronic searching will be implemented on databases of Nature, Science Online, PubMed, the Cochrane Library, MEDLINE, WorldSciNet, EMbase, AMED, the Wanfang Databse, SinoMed, China Biology Medicine Disc (CBMdisc), China National Knowledge Infrastructure (CNKI), the Chongqing VIP, and Chinese Science and Technology Periodical Database, with the temporal from the inception of database to September 21, 2020. For ongoing RCTs, researchers will immediately contact the author to obtain the latest clinical trial data for the data integrity of ongoing RCTs. A search strategy of the combination of text words and Medical Subject Headings (MeSH) terms will be adopted. The search strategy for PubMed database lists in Table 1.

**Table 1:** Search strategy for the PubMed database.

Number	Search Terms
1	Subacute thyroiditis. Mesh.
2	Subacute thyroiditis. ti, ab.
3	Subacute Thyroiditides. ti, ab.
4	Subacute Painful Thyroiditis. ti, ab.
5	Granulomatous Thyroiditis. ti, ab.
6	Subacute Nonsuppurative Thyroiditis. ti, ab.
7	De Quervain Thyroiditis. ti, ab.
8	Giant Cell Thyroiditis. ti, ab.
9	1 or 2–9
10	Xiaochaihu decoction. Mesh.
11	Xiaochaihu decoction. ti, ab.
12	XCHD. ti, ab.
13	Xiao-chai-hu-tang. Mesh.
14	Xiao-chai-hu-tang. ti, ab.
15	xiaochaihutang. ti, ab.
16	xiaochaihu-tang. ti, ab.
17	TJ9. ti, ab.
18	TJ-9. ti, ab.
19	XCHT herbal formula. ti, ab.
20	Shosaiko-to. ti, ab.
21	Shosaiko-toh. ti, ab.
22	Sho-saiko-to. ti, ab.
23	10 or 10−22
24	Randomized controlled trial. Mesh.
25	Randomized controlled trial. pt
26	Controlled clinical trail. Mesh.
27	Controlled clinical trail. pt
28	Clinical trial. pt
29	Random allocation. Mesh.
30	Random allocation. ti, ab
31	Randomly.ti, ab
32	Randomized. ti, ab
33	Double-blind method. Mesh.
34	Double-blind method. ti, ab
35	Single-blind method. Mesh.
36	Single-blind method. ti, ab
37	24 or 25-37
38	9 and 23 and 37

## 4 Data collection and analysis

### 4.1 Study selection

**Figure 1:**
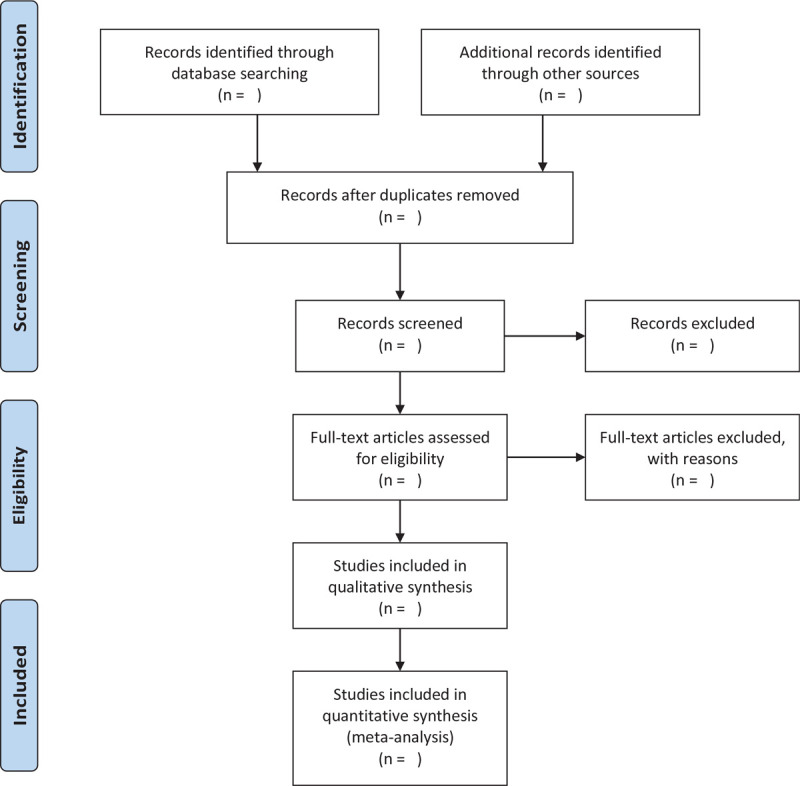
The flowchart of study selection.

After independently extracting the information from literature included in the study, 2 researchers will input the extracted information into a unified statistical table of data. Duplicate records and ineligible studies will be first eliminated, and then the full text of those eligible ones will be reviewed to confirm their compliance to the abovementioned inclusion criteria. If the 2 researchers cannot come to an agreement, the third researcher will make the final judgement. All the articles will be reviewed by the third author. A flowchart to show the whole process of study selection (Fig. 1).

### 4.2 Data extraction and management

According to Cochrane Handbook for Systematic Reviews of Interventions for inclusion characteristics study,^[31]^ the information extracted from each study include: the first author, the reference ID, year of publication, the number of included cases, age and gender of patients, randomization, allocation concealment method, blinding method, interventions and control measures, intervention and control groups sample size, intervention time, measures of outcome, primary outcomes, period of observation, follow-up duration, routine examination of safety. The researchers will contact the corresponding author of study in the case of insufficient reported data. If negotiation cannot help reach an agreement on data extraction, the final judgement will be handed to a third researcher.

### 4.3 Risk of bias assessment

Two researchers will independently evaluate the quality of literature included in the study, according to the Cochrane collaboration risk-of-bias assessment. Random sequence generation, allocation concealment, blinding, selective reporting, incomplete outcome data as well as other possible biases are included in the assessments. Related standards will be considered to classify risk of bias into 3 levels: high risk, low risk, and unclear risk. The discrepancies will be resolved by discussion and the third author will make the final judgement if the 2 researchers cannot reach an agreement.

### 4.4 Treatment effect measures

Odds ratio (OR) and risk ratio (RR) with 95% confidence interval (CI) will be used in measuring the treatment effects specific to dichotomous outcomes. For continuous outcomes, mean difference (MD) or standardized mean difference (SMD) with 95% CI will be calculated to measure the treatment effects.

### 4.5 Unit of analysis issues

The data of patients in RCTs will be adopted. Where more than 1 CXHD group is arranged in an RCT, multiple meta-analysis will be separately performed for each treatment group. For crossover trials, we will use data obtained from the first sequence. If there are multiple control measures, the intervention and control groups will be analyzed by summarizing all controls results.

### 4.6 Missing data management

The reason for missing data lost during data screening and extraction period will be identified. After the above audit is completed, the missing data will be obtained by contacted corresponding author. If missing data unable to be obtained, only available data will be analyzed and the effect and reason of such exclusion will be explained.

### 4.7 Heterogeneity assessment

A random- effects or fixed- effects model will be used for meta- analysis. Cochrane Handbook for Systematic Reviews of Interventions describes that the Higgins*I*^2^ statistic, a heterogeneity *x*^*2*^ test and a visual check of the forest plot can all help to assess the heterogeneity.^[32,33]^ A fixed-effects model will be used to pool the data with *P* value over .10 and the *I*^2^ value less than 50%. A random-effects model will be adopted in other cases. When a set of studies exhibit an obvious heterogeneity, factors leading to the heterogeneity, such as the characteristics of patients and degree of variation in interventions, will be discussed. The heterogeneity will be evaluated by the subgroup analysis or the sensitivity group if applicable.

### 4.8 Reporting bias assessment

The reporting biases will be assessed by a funnel plot if the meta-analysis includes over 10 trials. The asymmetry of the funnel plot will be evaluated by the Egger and Beggers tests. Meanwhile, *P* value less than .05 will be considered that the publication bias is significant.

### 4.9 Data analysis

The RevMan 5.3 software (Copenhagen: The Nordic Cochrane Centre, The Cochrane Collaboration, 2014) will be used for data analysis. The heterogeneity degree will help to confirm whether a random-effects model or a fixed-effects model will be adopted. The categorical variables will use the index of RR or OR with 95% CI. Continuous variables will adopt the index of MD or SMD with 95% CI. Chi-Squared distribution test and *I*^2^ statistic will be used to analysis the heterogeneity of included studies. If quantitative synthesis is not appropriate due to substantial heterogeneity, only qualitative analysis will be performed. In certain case, the subgroup analysis shall consider each subgroup carefully.

### 4.10 Subgroup analysis

Subgroup analysis will consider the heterogeneity exhibited by the phases of SAT progress (acute inflammatory phase, the second transient phase and the third phase), the dosage of XCHD, duration of treatment, measures in control groups.

### 4.11 Sensitivity analysis

In order to test if review conclusions are robust, primary outcomes will receive a sensitivity analysis based on criteria involving the size of sample, the quality of heterogeneity and the statistic model (whether it is a random-effects model or a fixed-effects model).

### 4.12 Grading the evidence quality

The evidence quality for obtained outcomes will be assessed by the GRADE method.^[34]^ After the assessment of certainty assessment, number of patients, effect, certainty and importance, evidence will be divided into 4 levels: high risk, moderate risk, low risk, and very low risk.

### 4.13 Ethics and dissemination

Meta-analysis is a secondary study of the published data, thus there is no need for obtaining the ethical approval or patients informed consent. The results will be published in journals reviewed by peers.

## 5 Discussion

Subacute thyroiditis is the most common painful thyroid disease and is a common cause of thyrotoxicosis.^[3]^ Treatment of SAT is based upon observational studies and clinical experience, with treatment directed towards relieving pain and hyperthyroid symptoms. NSAIDs have shown positive outcomes and corticosteroids have a role in severe cases.^[35]^ However, there is no evidence to suggest that NSAIDs and glucocorticoid therapy can reduce the incidence of hypothyroidism. Some patients will develop permanent hypothyroidism. Meanwhile, the side effects associated with the NSAIDs and glucocorticoids may occur. Adverse events caused by drugs lead to high treatment discontinuation rate, unsatisfying curative efficacy, and a high recurrence rate.

TCM is used to treat disease rather than only symptoms. Traditional Chinese medicine resource is a treasure house that contains many effective methods that have not been proven by modern science. Chinese herbal medicines may be a reasonable and safe alternative to treat SAT but there is not even a systematic evaluation of Chinese herbal medicines for SAT.^[21]^ Among the abundant Chinese herbal medicines, XCHD has been proven to be effective in the treatment of SAT.^[36]^ However, its real efficacy and safety are not well understood. Therefore, this study will evaluate the efficacy and safety of XCHD in treating SAT by searching and analyzing relevant studies comprehensively. In this study, we would draw a scientific conclusion and provide evidence of EBM for XCHD in the clinical treatment of SAT.

## Author contributions

**Conceptualization:** LinZhi Li, Rensong Yue.

**Data curation:** LinZhi Li, Lihong Zeng, Shengnan Wang.

**Formal analysis:** LinZhi Li, Lihong Zeng, Wuhui Zhuo.

**Funding acquisition:** Rensong Yue.

**Methodology:** LinZhi Li, Lihong Zeng, Yingying Sun.

**Project administration:** LinZhi Li.

**Resources:** Lihong Zeng, Shengnan Wang, Wuhui Zhuo.

**Software:** Yingying Sun.

**Writing – original draft:** LinZhi Li.

**Writing – review & editing:** Rensong Yue.
